# Prevalence of stress, anxiety and depression and their association with coping mechanisms among primary healthcare doctors in Petaling District, Selangor, during the transition to the endemic phase of COVID-19: A cross-sectional study

**DOI:** 10.51866/oa.686

**Published:** 2026-03-06

**Authors:** Abdul Rahim Ilyas, Mohamad Senin Muʼizuddin, Mohamad Nazir Syafiq, Augustina Chie Ing Wong, Hussin Sakina, Karunakaran Parimala, Abdul Hadi Said, Salziyan Badrin

**Affiliations:** 1 Klinik Kesihatan Seri Kembangan, Jalan Besar, Taman Muhibbah, Seri Kembangan, Selangor, Malaysia.; 2 Klinik Dr Muiz, G-1-56 & G-2-56, Jalan SP 11/3, Seri Pristana, Sungai Buloh, Selangor, Malaysia.; 3 Klinik Kesihatan Kota Damansara, Jalan Pekaka 8/3, Kota Damansara, Petaling Jaya, Selangor, Malaysia.; 4 Klinik Uzma Elmina, 7-G & 9-G, Jalan Eserina AA U16/AA Elmina East, Seksyen U16, Shah Alam, Selangor, Malaysia.; 5 Klinik Oasis Ara Damansara, A-G-03, Block A, No.2, Oasis Square, Jalan PJU 1A/7, Petaling Jaya, Selangor, Malaysia.; 6 Department of Family Medicine, Kulliyyah of Medicine, International Islamic University Malaysia, Kuantan, Pahang, Malaysia.; 7 Department of Family Medicine, School of Medical Sciences, Universiti Sains Malaysia Kubang Kerian, Kota Bharu, Kelantan, Malaysia.

**Keywords:** COVID-19, Depression, Anxiety, Coping skills, Primary healthcare

## Abstract

**Introduction:**

Following Malaysia’s transition to the endemic phase of COVID-19, primary healthcare doctors (PHDs) experienced a substantial increase in workload , which combined with workforce shortages and continued exposure to infection risk, contributed to sustained psychological distress among PHDs. Assessing the mental health status and coping strategies of healthcare professionals during the recovery phase is essential to ensure the continuity of national healthcare services.

**Methods:**

This cross-sectional study assessed the prevalence of stress, anxiety and depression among PHDs in private and public primary care clinics in Petaling, Selangor. It also analysed the relationship between these mental health conditions and coping mechanisms over 5 months. Selfadministered questionnaires containing sociodemographic items, as well as English versions of the Depression Anxiety Stress Scale-21 (DASS-21) and Brief COPE were utilised. Multiple logistic regression was applied to determine the associated factors of depression, anxiety and stress.

**Results:**

A total of 230 participants were included. The median age was 35 years. The prevalence of depression, anxiety and stress was 28.7%, 23.0% and 20.4% respectively. Workplace conflict was significantly associated with higher odds of depression (AOR=2.497; 95% CI=1.230–5.070), anxiety (AOR=5.337; 95% CI=2.329-12.232) and stress (A0R=3.078; 95% CI=1.339-7.075). Avoidant coping strategies were associated with higher odds of depression (AOR=2.743; 95% CI=1.150-6.544) and anxiety (AOR=5.776; 95% CI=2.010-16.598).

**Conclusion:**

Current trends underscore the need to implement evidence-based coping strategies that directly address underlying factors contributing to depression and anxiety. Additional targeted interventions are required to improve the mental health of physicians post COVID-19 pandemic.

## Introduction

COVID-19 is a novel infectious disease that has spread rapidly worldwide since late 2019 and was declared a pandemic by the World Health Organisation (WHO) on 11 March 2020. Malaysia, similar to many countries, experienced profound health system and psychosocial impacts. As of18 May 2024, Selangor – the most populous state – had reported 1,614,011 cases, with a prevalence of 10.5 cases per 100,000 people.^1^ When Malaysia entered the endemic phase on 1 April 2022, primary healthcare services resumed normal operations. Clinics saw an increase in outpatients, faced a backlog in managing chronic diseases and dealt with additional administrative work, all while still operating with a short-staffed workforce. These issues, along with ongoing worries about infection, kept stress levels high for primary healthcare doctors (PHDs).^2^ The Ministry of Health’s postpandemic recovery efforts also required PHDs to reintegrate routine preventive, maternal and chronic disease services that had been previously disrupted, thereby intensifying daily workloads.^3^

PHDs are the main points of contact in Malaysia’s healthcare system, playing important roles in both public and private sectors. They handle clinical care, administration and public health monitoring and oversee digital reporting systems that started during the pandemic.^4^ Because of their heavy workloads, high patient expectations and limited resources, they often face psychological strain and risk burnout. Several local studies during the acute pandemic period reported moderate-to-high levels of stress, anxiety and depression among healthcare workers. Still, most were hospital-based or conducted at the peak of the pandemic.^5,6^ A local study found that frontline primary healthcare workers experienced high levels of depression, anxiety and stress during the COVID-19 pandemic, underscoring their vulnerability to psychological distress.^7^ However, less is known about their mental health status as Malaysia transitioned into the endemic phase, when routine services resumed and workload pressures persisted.

The psychological well-being of doctors has significant implications for the quality of healthcare and the sustainability of the healthcare workforce. Ongoing stress, anxiety or depression can make it more difficult for doctors to pay attention, make good decisions and keep patients safe.^8^ In Malaysia, the number of doctors compared with the population has only recently improved to about 1 for every 420 people (or 2.38 doctors for every 1000 people).^9^ Supporting doctors’ mental health is essential to stop them from leaving their jobs and ensure patients keep receiving care in local clinics. Acting early on these mental health issues may help prevent long-term problems for both doctors and their patients.

Coping strategies play a vital role in determining how individuals respond to stress.^10^ Problem-focused coping has been shown to reduce emotional distress, whereas avoidant coping is often linked to poorer psychological outcomes.^5,7^ Identifying the types of coping mechanisms used by PHDs can guide the development of tailored interventions – such as stress-management training or institutional support programmes – to build resilience in this essential workforce.^1,7^

Therefore, this study aimed to determine the prevalence of stress, anxiety and depression and examine their association with coping mechanisms among PHDs in Petaling District, Selangor, during Malaysia’s transition to the endemic phase of COVID-19.

## Methods

### Study design and population

The study adopted a cross-sectional design, in which data were collected using a convenience sampling method from private and government PHDs from different areas of Petaling District, Selangor, from 1 April to 31 August 2023. Ethical approval was obtained from the Medical Research and Ethics Committee (MREC), Ministry of Health Malaysia, and the study was registered with the National Medical Research Register (NMRR ID-22-02896-8KF).

The study population included fully registered PHDs who had been practising in primary care settings either in public or private clinics or in university health clinics.

The independent variables assessed in this study included sociodemographic, occupational and personal factors that were selected based on previous local studies examining mental health among healthcare workers.^7^

### Sociodemographic variables

Ethnicity was categorised as Malay and non-Malay, according to participants’ self-identification. Marital status was self-reported and categorised as single (never married), married (legally married or living with a spouse) or divorced (legally separated or divorced at the time of the survey). Sex was classified as male and female and religion as Muslim and non-Muslim. Household income was recorded as a numerical variable representing the total net monthly income in Malaysian ringgit (MYR) of all earning members within participants’ household. For married participants, this included the combined income of the participant and their spouse. For single participants, it included their own income plus that of any cohabiting family members or housemates who contributed to shared household expenses. Net income was defined as total earnings, inclusive of allowances, perks and overtime pay, minus statutory deductions such as income tax, Employer Provident Fund and other miscellaneous contributions, based on the most recent payslip at the time of survey completion.

The number of dependents referred to the total number of individuals financially supported by participants. Dependents were defined as non-working immediate family members, including spouse and children, as well as other relatives such as parents or grandparents, for whom participants were partially or fully responsible for daily living expenses or upkeep.

The highest educational level referred to the most advanced academic or professional qualification that participants had completed and received formal recognition for. It was categorised as follows:

- Undergraduate qualification: Basic medical degree – Bachelor of Medicine, Bachelor of Surgery, Doctor of Medicine or equivalent.
- Postgraduate diploma: Graduate Certificate in Family Medicine, Diploma in Family Medicine, Diploma in Dermatology, Diploma in Occupational Safety and Health or other equivalent postgraduate diplomas.
- Postgraduate qualification: Advanced Training in Family Medicine, Fellowship of the Royal Australian College of General Practitioners, Master’s degrees (e.g. Master of Family Medicine, Master of Public Health and Master of Clinical Administration or Clinical Sciences) and doctoral-level qualifications such as Doctor of Public Health.

### Occupational variables

Profession was classified according to participants’ designation and postgraduate training status in primary care. Primary care doctors (PCDs) who had completed formal postgraduate training in family medicine were categorised as family medicine specialists (FMSs). Those without such specialisation were classified as either medical officers (MOs) if practising in public or university health clinics or general practitioners (GPs) if practising in private clinics. This classification was adapted from previous local studies on primary care workforce characteristics.^7^

Working experience was defined as the total number of years spent working at the primary care clinic (excluding housemanship training and previous hospital service). The average number of patients seen per day, as reported by participants, was compared with their self-reported average total number of patients attended to daily at their principal place of practice. This included both COVID-19 and non-COVID-19-related cases, encompassing walk-in and appointment-based consultations.

The number of working hours per day referred to the total duration of time spent at participants’ principal place of practice each working day, including lunch hour. It was categorised as less than 9 hours or 9 hours or more. The current working sector was described as the principal place of practice. It was categorised as either government (public health or university-based clinics) or private (privately owned primary care clinics).

Overtime jobs referred to any additional clinical or administrative duties performed beyond participants’ regular working hours during the transition to the endemic phase of COVID-19 (beginning 1 April 2022). This included both voluntary and employer-mandated additional duties, such as locum sessions, extended clinic hours, COVID-19 Assessment Centre services or other after-hours clinical activities. The options included yes (performed overtime work) and no (did not perform overtime work).

Experience in managing COVID-19 was defined as having been directly involved in screening, diagnosing or managing patients with suspected or confirmed COVID-19 during the pandemic or the transition phase to endemicity. Workplace conflicts referred to any form of active opposition or disagreement experienced by participants with coworkers – including superiors, colleagues or subordinates – during the transition to the endemic phase of COVID-19 (beginning 1 April 2022). Conflicts could take various forms, such as verbal, physical, sexual, financial or psychological conflicts. Responses were categorised as yes (had experienced workplace conflict) or no (had not experienced workplace conflict).

Perception of increased work burden referred to the subjective assessment by participants that their workload had increased compared with the prepandemic period.

### Personal variables

Underlying medical illness was self-reported and included any chronic medical conditions that required regular medical follow-up. Financial hardship was defined as self-reported difficulty meeting monthly financial commitments or expenses. Family conflicts referred to any form of active opposition or disagreement experienced by participants with immediate family (spouse, parents or children) that caused psychological distress. Conflicts could take various forms, such as verbal, physical, sexual, financial or psychological conflicts. Responses were categorised as yes (had experienced family conflicts) or no (had not experienced family conflicts).

The dependent variables were depression, anxiety and stress, assessed using the Depression Anxiety Stress Scale-21 (DASS-21), as well as coping strategies, measured using the Brief COPE questionnaire.^11^

The DASS-2115 is a self-administered instrument consisting of 21 items that assess the negative emotional states of depression, anxiety and stress. Each of the three subscales contains seven items, rated on a 4-point Likert scale ranging from 0 *(did not apply to me at all)* to 3 *(applied to me very much or most of the time).*

The depression subscale measures dysphoria, hopelessness, devaluation of life, self-deprecation, lack of interest or involvement and anhedonia. The anxiety subscale assesses autonomic arousal, skeletal muscle effects, situational anxiety and subjective experience of anxious affect. The stress subscale evaluates difficulty relaxing, nervous arousal, irritability and being easily upset or overreactive.

Scores for each subscale are summed and multiplied by 2 to align with the full 42-item DASS scoring. Severity is interpreted according to the following standard DASS cut-off scores: depression: normal (0–9), mild (10–13), moderate (14–20), severe (21–27) and extremely severe (≥28); anxiety: normal (0–7), mild (8–9), moderate (10–14), severe (15–19) and extremely severe (≥20); and stress: normal (0–14), mild (15–18), moderate (19–25), severe (26–33) and extremely severe (≥34).

The DASS-21 has been validated in both English and Malay and has demonstrated strong internal consistency among Malaysian healthcare professionals.^16,17^

The Brief COPE questionnaire^11^ is a validated 28-item self-administered instrument developed to assess a wide range of coping responses to stress. Each item is rated using a 4-point Likert scale to quantify the frequency of coping behaviours, ranging from 1 *(I haven’t been doing this at all)* to 4 *(I’ve been doing this a* lot). The scale contains the following 14 subscales, with each subscale comprising two items that assess distinct coping strategies:

1. Active coping involves implementing specific actions to eliminate or mitigate the identified stressor.
2. Planning is about coming up with ways to deal with the stressor.
3. Positive reframing entails interpreting stressful events in a more constructive or optimistic manner.
4. Acceptance is about acknowledging what is happening and adjusting to it.
5. Humour means using jokes or a light-hearted attitude to cope with stress.
6. Religion is about finding comfort and meaning through spiritual or religious activities.
7. Using emotional support means receiving empathy, understanding or encouragement from others.
8. Instrumental support means reaching out to others for advice, help or information.
9. Self-distraction involves doing activities that help take the mind off what is causing stress.
10. Denial is when someone does not accept that the source of stress is real or tries to ignore it.
11. Venting means sharing negative feelings that arise because of stress.
12. Substance use refers to turning to alcohol, drugs or other substances to cope with distress.
13. Behavioural disengagement happens when someone stops trying to deal with what is causing stress.
14. Self-blame is when a person blames themselves for causing or not stopping the stressful situation.

For analysis, the subscales were grouped into three main coping domains, consistent with previous studies:

- Problem-focused coping: active coping, planning and use of instrumental support.
- Emotion-focused coping: positive reframing, acceptance, religion, use of emotional support and humour.
- Avoidant-focused coping: self-distraction, denial, venting, substance use, behavioural disengagement and self-blame.

Higher scores within each domain indicate more frequent use of that coping strategy. The English/ Malay versions of the Brief COPE have been used and validated in Malaysian populations, including nurses and other healthcare workers. Previous national and local studies have successfully employed the Brief COPE to assess coping strategies among Malaysian healthcare staff. Therefore, the Brief COPE was considered appropriate for evaluating coping responses in this study.^12-14^

A total of 1242 PHDs working in both private and government clinics in Petaling District, Selangor, were identified. This figure was officially obtained from the Private Medical Practice Control Section (CKAPS) of the Selangor Health Department for private GPs and the Petaling District Health Office (PKD Petaling) for government clinic doctors.

### Sample size calculation

The estimated sample size of204 was calculated using the OpenEpi software version 3. According to a previous study conducted among frontline primary healthcare workers,^7^ the prevalence of depression, anxiety and stress was 19.7%, 15.2% and 2.8%, respectively. The sample size was calculated based on the prevalence of depression, as this yielded the highest number of participants. Given a non-response rate of 20%, the final sample size was estimated to be 244.

### Study tools, participant recruitment, sampling method and data collection

A Google Form link with a brief introduction of the study, a consent form and the questionnaire were disseminated through the organisations representing doctors such as the Malaysian Medical Association Selangor Branch, Academy of Family Physicians of Malaysia, Family Medicine Specialist Association, Petaling District Health Office and CKAPS, Selangor State Health Department with permission, via WhatsApp and email.

PHDs received invitations through the professional associations stated. Each invitation, sent via email and social media platforms, contained links to the study information and questionnaire. The number of times the link was sent was not standardised and depended on the internal communication schedule of each association.

The questionnaire consisted of three sections: sociodemographic questions, the DASS-21 and the Brief COPE.

- Inclusion criteria:
  - Doctors currently working full-time in primary care clinics (government and private clinics) in Petaling District, Selangor
    1. Government clinics: FMSs or MOs (on contract or permanent post) with an official placement at the clinic
    2. Private clinics: GPs working full-time as resident doctors at the clinic (locum doctors were excluded)
    3. Petaling District: includes areas served by the following city councils:
      - Shah Alam City Council (MBSA)
      - Petaling Jaya City Council (MBPJ)
      - Subang Jaya City Council (MBSJ)
  - Doctors working for at least 6 months before participation in this research
    - The 6 months were counted from the first day of reporting duty at the current workplace (without interruptions) to the date of survey completion. This criterion ensured that participants had adequate experience at their current workplace to provide informed responses.
  - Doctors able to understand and communicate in English
- Exclusion criteria:
  - Non-Malaysian doctors
  - Doctors on prolonged leave during data collection
    - Prolonged leave was defined as a period during which a participant was not actively working or practising at their principal workplace, lasting more than 3 months, including, but not limited to, annual leave, unpaid leave, study leave, maternity leave, sick leave or any equivalent leave.
  - Locum practitioners
  - Doctors already diagnosed with mental illness and/or started on psychiatric medication

Only those who fulfilled the abovementioned inclusion criteria and did not fall into any of the exclusion criteria were able to complete the Google Form.

### Statistical analysis

Data analysis was performed using the IBM Statistical Package for the Social Sciences (IBM Corp., Armonk, NY, USA) version 27.0. Chi-square tests followed by linear regression analysis were performed. A P-value of less than 0.05 was considered statistically significant.

## Results

**Figure 1:**
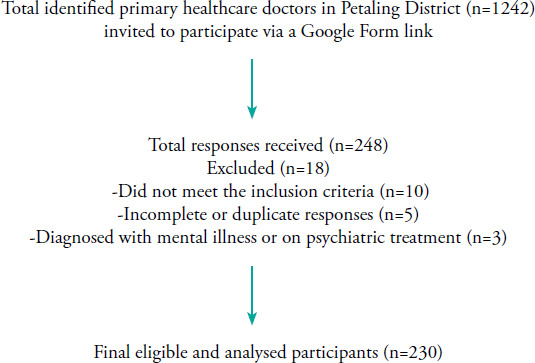
Flow chart of the study.

A total of 248 participants completed the survey. Eighteen participants were later found to be disqualified from the study; hence, only 230 responses were analysed (non-response rate of 7.3%), as presented in Figure 1.

Table 1 shows the sociodemographic characteristics of the participants. The median age was 35 years (IQR=7). The majority of the participants were women (74.3%), Malay (53.9%), Muslim (55.7%) and married (72.6%), with a median household income of MYR 10,000 (IQR=7000) and median working experience of 6 years (IQR=8). Additionally, most participants were undergraduates (72.6%).

**Table 1:** Sociodemographic characteristics of the participants.

Characteristic	n	Percentage	Median (IQR)
**Age (year)**			35 (7)
**Ethnicity**			
Malay	124	53.9	
Non-Malay	106	46.1	
**Marital status**			
Single	57	24.8	
Married	167	72.6	
Divorced	6	2.6	
**Sex**			
Male	59	25.7	
Female	171	74.3	
**Religion**			
Muslim	128	55.7	
Non-Muslim	102	44.3	
**Household income (MYR)**			10,000 (7000)
**Number of dependents**			2 (2)
**Educational level**			
Undergraduate	167	72.6	
Postgraduate diploma	38	16.5	
Postgraduate qualification	25	10.9	
**Profession**			
Family medicine specialist	12	5.2	
Medical officer	133	57.8	
General practitioner	85	37.0	
**Working experience (year)**			6 (8)
**Number of patients seen per day**			35 (20)
**Number of working hours per day**			
<9 hours	153	66.5	
>9 hours	77	33.5	
**Experience in managing COVID-19**			
Yes	212	92.2	
No	18	7.8	
**Underlying medical illness**			
Yes	42	18.3	
No	188	81.7	
**Working sector**			
Public	128	55.7	
Private	102	44.3	
**Personal history of COVID-19**			
Yes	185	80.4	
No	45	19.6	
**History of COVID-19-related death in the family**			
Yes	30	13	
No	200	87	
**Overtime job**			
Yes	128	55.7	
No	102	44.3	
**Financial hardship**			
Yes	96	41.7	
No	134	58.3	
**Family conflict**			
Yes	78	33.9	
No	152	66.1	
**Workplace conflict**			
Yes	106	46.1	
No	124	53.9	
**Perception of increase in work burden**			
Yes	198	86.1	
No	32	13.9	

Table 2 presents the prevalence of stress, anxiety and depression and the coping mechanisms used by the participants. The highest prevalence was noted for depression at 28.7%, followed by anxiety at 23% and stress at 20.4%. The coping mechanisms applied by the participants were divided into three categories, with avoidant-focused coping as the most prevalent at 39.15%, followed by problem-focused coping at 31.7% and emotion-focused coping at 29.1%.

**Table 2:** Prevalence of stress, anxiety and depression and coping mechanisms.

Variable	n (%)
**Stress**	
Yes	183 (79.6)
No	47 (20.4)
**Anxiety**	
Yes	177 (77.0)
No	53 (23.0)
**Depression**	
Yes	164 (71.3)
No	66 (28.7)
**Coping mechanism**	
Problem-focused	73 (31.7)
Emotion-focused	67 (29.1)
Avoidant-focused	90 (39.1)

As shown in Table 3, the bivariate analysis indicated that the variables significantly associated with depression included marital status, sex, household income, working experience, working sector, overtime job, financial hardship, family conflict, workplace conflict and coping mechanism. These 10 significant variables were entered into the multiple logistic regression (MLR) analysis (Table 4) to determine their actual association with depression after adjusting for confounders. The analysis revealed that only three variables were significantly associated with depression. Female sex was 2.7 times more likely to be associated with depression (AOR=2.662; 95% CI=1.111–6.376), while the presence of workplace conflicts was 2.4 times more likely to be associated with depression (AOR=2.497; 95% CI=1.230–5.070). Additionally, avoidant coping was the most common coping mechanism utilised to combat depression (AOR=2.743; 95% CI=1.150-6.544).

**Table 3:** Bivariate analysis of the factors associated with depression, stress and anxiety.

Factor	Depression				Anxiety				Stress			
	Yes	No	X^2^	P-value	Yes	No	X^2^	P-value	Yes	No	X^2^	P-value
**Age, year, median (IQR)^†^**	34.0 (7.0)	36.0 (6.0)		0.051	33.0 (8.0)	36.0 (7.0)		0.004^*^	33.0 (7.0)	36.0 (6.0)		0.012^*^
Ethnicity, n (%) Malay Non-Malay	38 (57.6%) 28 (42.4%)	86 (52.4%) 78 (47.6%)	0.5	0.48	33 (62.3%) 20 (37.7%)	91 (51.4%) 86 (48.6%)	1.933	0.164	26 (55.3%) 21 (44.7%)	98 (53.6%) 85 (46.4%)	0.047	0.828
Marital status, n (%) Married Unmarried	40 (60.6%) 26 (39.4%)	127.0 (77.4%) 37 (22.6%)	6.705	0.010^*^	33 (62.3%) 20 (37.7%)	134 (75.7%) 43 (24.3%)	3.706	0.054	28 (59.6%) 19 (40.4%)	139 (76.0%) 44 (24.0%)	5.046	0.025^*^
Sex, n (%) Male Female	10.0 (15.2%) 56.0 (84.8%)	49.0 (29.9%) 115.0 (70.1%)	5.351	0.021^*^	8 (15.1%) 45 (84.9%)	51 (28.8%) 126 (71.2%)	4.025	0.045^*^	6 (12.8%) 41 (87.2%)	53 (29.0%) 130 (71.0%)	5.143	0.023
Religion, n (%) Muslim Non-Muslim	39 (59.1%) 27 (40.9%)	89.0 (54.3%) 75 (45.7%)	0.443	0.505	34 (64.2%) 19 (35.8%)	94 (53.1%) 83 (46.9%)	2.016	0.156	27 (57.4%) 20 (42.6%)	101 (55.2%) 82 (44.8%)	0.077	0.781
Household income, MYR, median (IQR)t	9000(7345)	11,000 (6000)		<0.001^*^	9000 (7000)	10,000 (6800)		0.006^*^	9000 (9000)	10,000 (6600)		0.045^*^
Underlying medical illness, n (%) Yes No	12 (18.2%) 54 (81.8%)	30 (18.3%) 134 (81.7%)	0	0.984	12 (22.6%) 41 (77.4%)	30 (16.9%) 147 (83.1%)	0.885	0.347	9 (19.1%) 38 (80.9%)	33 (18.0%) 150 (82.0%)	0.031	0.860
Number of dependents, median (IQR)^†^	2.0(2.0)	2.0 (2.0)		0.193	2.0 (2.0)	2.0 (2.0)		0.737	2.0 (2.0)	2.0 (2.0)		0.536
Educational level, n (%) Undergraduate Postgraduate diploma Postgraduate qualification	48 (72.7%) 10 (15.2%) 8 (12.1%)	119 (72.6%) 28 (17.1%) 17 (10.4%)	0.239	0.887	40 (75.5%) 7 (13.2%) 6 (11.3%)	127 (71.8%) 31 (17.5%) 19 (10.7%)	0.549	0.760	37 (78.7%) 5 (10.6%) 5 (10.6%)	130 (71.0%) 33 (18.0%) 20 (10.9%)	1.545	0.462
Profession, n (%) Family medicine specialist Medical officer General practitioner	3 (4.5%) 45 (68.2%) 18 (27.3%)	9 (5.5%) 88 (53.7%) 67 (40.9%)	4.145	4.145	2 (3.8%) 29 (54.9%) 22 (41.5%)	10 (5.6%) 104 (58.8%) 63 (35.6%)	0.777	0.678	3 (6.4%) 32 (68.1%) 12 (25.5%)	9 (4.9%) 101 (55.2%) 73 (39.9%	3.315	0.191
Working experience, year, median (IQR)^†^	5.0(6.0)	7.0 (7.0)		0.040^*^	5.0 (7.0)	7.0 (7.0)		0.014^*^	4.0 (7.0)	7.0 (7.0)		0.026^*^
Number of patients seen per day, median (IQR)^†^	40 (20)	32.5 (18)		0.146	40 (28)	35 (10)		0.248	40 (20)	35 (15)		0.159
Number of working hours per day, n (%) <9 hours >9 hours	39 (59.1%) 27 (40.9%)	114 (69.5%) 50 (30.5%)	2.295	0.13	31 (58.5%) 22 (41.5%)	122 (68.9%) 55 (31.1%)	1.995	0.158	26 (55.3%) 21 (44.7%)	127 (69.4%) 56 (30.6%)	3.329	0.068
Experience in managing COVID-19, n (%) Yes No	58 (87.9%) 8 (12.1%)	154 (93.9%) 10 (6.1%)	2.367	0.124	45 (84.9%) 8 (15.1%)	167 (94.4%) 10 (5.6%)	5.044	0.025^*^	40 (85.1%) 7 (14.9%)	172 (94.0%) 11 (6.0%)	4.090	0.043^*^
Working sector, n (%) Public Private	44 (66.7%) 22 (33.3%)	84 (51.2%) 80 (48.8%)	4.55	0.033^*^	27 (50.9%) 26 (49.1%)	101 (57.1%) 76 (42.9%)	0.619	0.432	30 (63.8%) 17 (36.2%)	98 (53.6%) 85 (46.4%)	1.601	0.206
Personal history of COVID-19, n (%) Yes No	53 (80.3%) 13 (19.7%)	132 (80.5%) 32 (19.5%)	0.01	0.975	43 (81.1%) 10 (18.9%)	142 (80.2%) 35 (19.8%)	0.021	0.884	40 (85.1%) 7 (14.9%)	145 (79.2%) 38 (20.8%)	0.819	0.365
History of COVID-19-related death in the family, n (%) Yes No	9 (13.6%) 57 (86.4%)	21 (12.8%) 143 (87.2%)	0.29	0.866	6 (11.3%) 47 (88.7%)	24 (13.6%) 153 (86.4%)	0.180	0.671	5 (10.6%) 42 (89.4%)	25 (13.7%) 158 (86.3%)	0.301	0.583
Overtime job, n (%) Yes No	45 (62.8%) 21 (31.8%)	83 (50.6%) 81 (49.4%)	5.888	0.015^*^	36 (67.9%) 17 (32.1%)	92 (52.0%) 85 (48.0%)	4.203	0.04^*^	32 (68.1%) 15 (31.9%)	96 (52.5%) 87 (47.5%)	3.700	0.054
Financial hardship, n (%) Yes No	37 (56.1%) 29 (49.3%)	59 (36%) 105 (64%)	7.807	0.005^*^	30 (56.5%) 23 (43.4%)	66 (37.3%) 111 (62.7%)	6.258	0.012^*^	27 (57.4%) 20 (42.6%)	69 (37.7%) 114 (62.3%)	5.993	0.014^*^
Family conflict, n (%) Yes No	36 (54.5%) 30 (45.5%)	42 (25.6%) 36 (54.5%)	17.581	<0.001^*^	31 (58.5%) 22 (41.5%)	47 (26.6%) 130 (73.4%)	18.562	<0.001^*^	28 (59.6%) 19 (40.4%)	50 (27.3%) 133 (72.7%)	17.356	<0.001^*^
Workplace conflict, n (%) Yes No	45 (68.2%) 21 (31.8%)	61 (37.2%) 103 (62.8%)	18.186	<0.001^*^	41 (77.4%) 12 (22.6%)	65 (36.7%) 112 (63.3%)	27.105	<0.001^*^	36 (76.6%) 11 (23.4%)	70 (38.3%) 113 (61.7%)	22.129	<0.001^*^
Perception of increase in work burden, n (%) Yes No	63 (95.5%) 3 (4.5%)	135 (82.3%) 29 (17.7%)	6.781	0.009	49 (92.5%) 4 (7.5%)	149 (84.2%) 28 (15.8%)	2.330	0.127	47 (100.0%) 0 (0.0%)	151 (82.5%) 32 (17.5%)	9.547	0.002^*^
Coping mechanism, n (%) Problem-focused Emotion-focused Avoidant-focused	13 (19.7%) 23 (34.8%) 30 (45.5%)	60 (36.6%) 44 (26.8%) 60 (36.6%)	6.214	0.045^*^	8 (15.1%) 19 (35.8%) 26 (49.1%)	65 (36.7%) 48 (27.1%) 64 (36.2%)	8.813	0.012^*^	12 (25.5%) 15 (31.9%) 20 (42.6%)	61 (33.3%) 52 (28.4%) 70 (38.3%)	1.051	0.591

* P<0.05, statistically significant

† Values are presented as median (IQR); comparisons were performed using the Mann–Whitney U test. IQR = interquartile range.

**Table 4:** Multiple logistic regression analysis of the factors associated with depression, anxiety and stress.

Factor	Depression				Anxiety				Stress			
	Adjusted odds ratio	95% CI	d.f.	P-value	Adjusted odds ratio	95% CI	d.f.	P-value	Adjusted odds ratio	95% CI	d.f.	P-value
Age, year, median (IQR)					0.876	0.757-1.1012	3.219	0.073	0.885	0.766-1.024	2.691	0.101
Marital status, n (%) Married Unmarried	1 1.769	0.806-3.880	2.026	0.155					1 1.693	0.707-4.057	1.395	0.238
Sex, n (%) Male Female	1 2.662	1.111-6.376	4.825	0.028^*^	1 2.835	1.004-8.005	3.587	0.049^*^	1 2.811	0.982-8.050	3.707	0.054
Household income, MYR, median (IQR)	1	1-1	1.909	0.167	1.000	1-1	0.342	0.559				
Working experience, year, median (IQR)	0.956	0.886-1.032	1.342	0.247	1.015	0.879-1.174	1.015	0.836	1.019	0.882-1.178	0.067	0.795
Experience in managing COVID-19, n (%) Yes No					1 6.114	1.171-21.861	7.758	0.005^*^	1 6.588	1.769-24.530	7.899	0.005^*^
Working sector, n (%) Public Private	1.771 1	0.877-3.576	2.541	0.111								
Overtime job, n (%) Yes No	1.485 1	0.733-3.009	1.202	0.273	1.148 1	0.525-2.510	0.120	0.729				
Financial hardship, n (%) Yes No	1.848 1	0.875-3.903	2.592	0.107	1.494 1	0.667-3.345	0.953	0.329	1.307 1	0.587-2.912	0.430	0.512
Family conflict, n (%) Yes No	1.729 1	0.826-3.622	2.108	0.147	2.280 1	1.003-5.183	3.870	0.049^*^	2.237 1	0.981-5.102	3.661	0.056
Workplace conflict, n (%) Yes No	2.497 1	1.230-5.070	6.410	0.011^*^	5.337 1	2.329-12.232	15.661	<0.001^*^	3.078 1	1.339-7.075	7.006	0.008^*^
Perception of increase in work burden, n (%) Yes No								0.000	0	0	0.998
Coping mechanism, n (%) Problem-focused Emotion-focused Avoidant-focused	1 2.028 2.743	0.859-4.788 1.150-6.544	5.177 2.603	0.107 0.023^*^	1 3.701 5.776	1.335-10.265 2.010-16.598	10.917 6.322 10.605	0.004^*^ 0.012^*^ 0.001^*^				

* P<0.05, statistically significant

For anxiety, the significant variables identified in the bivariate analysis (Table 3) were age, sex, household income, working experience, experience in managing COVID-19, overtime jobs, financial hardship, family conflicts, workplace conflicts and coping mechanisms. Among these 10 variables, only five were found to be significantly associated with anxiety when entered into the MLR analysis (Table 4). These variables were female sex (AOR=2.835; 95% CI=1.004–8.005), no experience in managing COVID-19 (AOR=6.1l4; 95% CI=1.171–21.861), the presence of family conflicts (AOR=2.280; 95% CI=1.003–5.183), the presence of workplace conflicts (AOR=5.337; 95% CI=2.329–12.232) and all three coping mechanisms (i.e. problem-[P=0.04], emotion-[AOR=3.701; 95% CI=1.335–10.265] and avoidant-focused coping [AOR=5.776; 95% CI=2.010-16.598]).

The significant variables associated with stress identified in the bivariate analysis (Table 3) were age, marital status, sex, household income, working experience, experience in managing COVID-19, financial hardship, family conflicts, workplace conflicts and perception of increased work burden. These 10 variables were entered into the MLR analysis (Table 4) to determine their actual association with stress while adjusting for confounders. Among them, only the following two were found to be significantly associated with stress: no experience in managing COVID-19 (AOR=6.588; 95% CI=1.769–24.530) and the presence of workplace conflicts (AOR=3.078; 95% CI=1.339-7.075).

## Discussion

### Prevalence of depression, anxiety and stress

In the present study, the prevalence of depression, anxiety and stress among the PCDs in Petaling District, Selangor, was 28.7%, 23.0% and 20.4%, respectively. These findings indicate a considerable level of psychological distress among doctors, even during the transition to the endemic phase of COVID-19.

The prevalence found in our study is higher than the rates reported by Salaton and Bulgiba,^7^ who found lower rates of depression (19.7%), anxiety (15.2%) and stress (2.8%) among healthcare doctors in Selangor during the pandemic phase using the PHQ-9 and GAD-7. This difference in prevalence may be attributed to variations in screening tools, as the DASS-21 used in our study is more sensitive to co-existing symptoms of stress, anxiety and depression, whereas the PHQ-9 and GAD-7 are domain-specific. Furthermore, our study focused on PCDs in Petaling District, a smaller and more urbanised population compared with the state-wide cohort studied by Salaton and Bulgiba.

Comparable local studies using the DASS-21 reported similar or slightly higher prevalence rates. For instance, a survey among Malaysian healthcare shift workers in Klang Valley revealed that 40.7% had at least one symptom of depression, anxiety or stress during the pandemic.^18^ Another study among medical interns in Malaysia found prevalence rates of 26.2%, 39.9% and 29.7% for depression, anxiety and stress, respectively.^19^ These studies, which used the same tool, suggest that our observed prevalence falls within the expected range for Malaysian healthcare professionals, particularly during or following the pandemic period.

However, studies among patients attending primary care clinics in Klang Valley reported lower rates of depression (11.5%), anxiety (30.5%) and stress (12.5%) using the DASS-21.^20^ This highlights the need for further research, as PCDs may experience a greater psychological burden compared with the general population owing to occupational stressors and the demands of clinical practice.

Similarly, a study among hospital emergency MOs in Malaysia showed lower rates of depression (10.7%), anxiety (28.6%) and stress (7.9%),^21^ despite comparable exposure to clinical pressure. This suggests that the distinct administrative and relational challenges in primary care settings may also contribute to heightened distress levels among primary care physicians.

### Factors associated with mental distress

In this study, several sociodemographic and occupational factors were associated with symptoms of depression, anxiety and stress among the PCDs. Family conflict was strongly associated with higher odds of depression, anxiety and stress. Family dynamics have been reported to be negatively correlated with depressive symptoms, while poor family intimacy or emotional expression can trigger depressive episodes among young adults.^22^ This highlights the importance of a supportive home environment in maintaining mental health, particularly among healthcare professionals facing high occupational demands.

Female sex was significantly associated with higher levels of depression and anxiety, consistent with previous reports among healthcare workers during the COVID-19 pandemic.^23-26^ Women may have greater physiological and psychological vulnerability to emotional stress due to hormonal fluctuations and a higher sensitivity to interpersonal relationships. In addition, specific forms of depression and anxiety, such as premenstrual dysphoric disorder, postpartum depression and postmenopausal anxiety disorders, may contribute to the observed sex differences.^27^

Having experience in managing COVID-19 cases during the transition to the endemic phase was independently associated with a lower risk of anxiety and stress. Although earlier studies conducted during the acute pandemic phase reported higher psychological distress levels among healthcare workers directly involved in treating patients with COVID-19,^26,27^ the present finding suggests an adaptive response. Prolonged exposure to high-stress situations during the pandemic may have strengthened the coping mechanisms and psychological resilience of the PHDs, leading to lower distress levels once the stressors were removed. Similarly, Ofei-Dodoo et al. reported that family physicians who managed patients with presumptive or confirmed COVID-19 during the pandemic phase experienced greater stress levels,^28^ supporting the notion that early exposure to crises can subsequently facilitate better adjustment in later stages of recovery.

Our research revealed that workplace conflict was also a significant predictor of mental distress. The doctors who reported conflicts at the workplace were 2.5, 5.4 and 3 times more likely to experience depression, anxiety and stress, respectively, than those without such conflicts. Workplace conflict may involve strained relationships among colleagues, including support staff, supervisors and administrative staff, which can impair team cohesion and lead to increased emotional exhaustion among staff members. In the study by Kakemam et al., high levels of depression, anxiety and stress among Iranian primary healthcare workers were linked to organisational policies, work conditions and job-related stressors.^29^ Likewise, Zhang et al. demonstrated that work-family conflict was significantly associated with depression among healthcare workers in the northeastern United States,^30^ and Liang et al. found a positive correlation between work-family conflict and anxiety among hospital-based healthcare workers in China.^31^ These findings underscore the vital role of healthcare administrators, mental health professionals, policymakers and researchers in fostering healthy workplace relationships and implementing effective conflict management systems within primary care settings.

### Association of depression, anxiety and stress with coping mechanisms

Our study showed that during the transition to the endemic phase of COVID-19, the avoidant coping strategy was the most common coping mechanism utilised by the PHDs in Petaling District, Selangor, to combat depression and anxiety. These doctors were 2.7 times more likely to opt for an avoidant coping mechanism than a problem-focused coping mechanism. These findings suggest that healthcare workers may gravitate towards avoidance coping mechanisms due to the perceived immediate relief they provide from distressing situations. Additionally, the stigma surrounding mental health issues in healthcare settings and the demanding nature of the profession may contribute to the preference for avoidance coping as a way to maintain a façade of competence and professionalism. While avoidance coping may offer temporary relief, it is generally less effective in addressing the underlying causes of depression and anxiety than problem-and emotion-focused coping strategies. Problem-focused coping allows healthcare workers to actively address the causes of their distress, while emotion-focused coping helps them regulate their emotional responses and seek support from others. Problem- and emotion-focused coping strategies have been associated with better mental health outcomes, including reduced levels of anxiety and depression.^32^ In contrast, avoidance coping strategies tend to be less effective and may even exacerbate psychological distress over time.^33^

Our study found no association between stress and coping mechanisms. This indirectly reflects the resilience and ability of PCDs to cope with stressful conditions following the pandemic crisis.

### Study limitations and strengths

Several limitations should be considered when interpreting our results. Our focus on PCDs in Petaling District, Selangor, means the findings may not be representative of all healthcare providers in Malaysia or generalisable to other settings. The reliance on self-reported questionnaires (the DASS-21 and Brief COPE) also carries a risk of self-reporting bias, as the participants may have provided socially desirable answers about their mental health and coping strategies. Furthermore, the study’s cross-sectional design captures data at a single point, which limits our ability to establish cause-and-effect relationships. Finally, the use of convenience sampling through professional networks may have led to selection bias, as doctors with a particular interest in mental health or greater availability were more likely to respond.

Despite these limitations, the study offers valuable insights. It is one of the few local investigations conducted as the country transitioned into the COVID-19 endemic phase, providing a timely snapshot of the psychological well-being of frontline doctors as they adapted to postpandemic challenges. The use of established instruments (the DASS-21 and Brief COPE) allows for a reliable comparison with other research. By examining a wide array of sociodemographic, occupational and psychosocial factors, our analysis was able to pinpoint key predictors of depression, anxiety and stress among this vital workforce.

## Conclusion

Several factors such as age, sex, experience in managing COVID-19 cases and family and workplace conflicts were significantly associated with the development of poor mental health among PHDs during the endemic phase of COVID-19.

Workplace culture interventions and self-care are crucial for addressing the social deficit and enhancing the quality of life and cognitive function of healthcare professionals following the COVID-19 pandemic. Fostering a culture that encourages and supports problem- and emotion-focused coping among healthcare workers is essential for their well-being and the quality of care they provide.

## Data sharing statement

The raw and analysed datasets of the current study are available from the corresponding author upon request.

**How does this paper make a difference in general practice?**

- This paper reveals the prevalence of stress, anxiety and depression and their association with coping mechanisms among primary healthcare doctors in Petaling District, Selangor, during the transition to the endemic phase of COVID-19.
- The findings provide valuable information and direction to guide relevant authorities (employers and the Ministry of Health) in developing policies to create a healthier and more supportive workplace for doctors, as well as to inform future studies, particularly in areas related to the psychological health of healthcare workers.
